# Building Faculty Performance Evaluation from the Ground-Up: Designing Tools and Gauging Acceptance

**DOI:** 10.1007/s40596-025-02192-w

**Published:** 2025-09-04

**Authors:** Kelly L. Cozza, Althea A. Scott, Elle S. Cleaves, Derrick A. Hamaoka, Courtney C. O’Keefe, Eungjae Kim, Matthew Hawks

**Affiliations:** 1 Uniformed Services University of the Health Sciences, Bethesda, MD USA; 2 Brooke Army Medical Center, University of Texas Health, San Antonio, TX USA; 3 Walter Reed National Military Medical Center, Bethesda, MD USA

Students should have the opportunity to assess their clinical faculty’s performance and provide feedback in a way similar to how faculty assess student performance, best obtained at the time of their rotation [1–5]. Faculty development is a valuable activity that should help clinician-educators better understand their role [6], impact their ability to provide high-quality and relevant feedback to their students, and improve student and faculty performance. The Standards for Accreditation of Medical Education Programs Leading to the Medical Doctor Degree of the Liaison Committee on Medical Education (LCME) require faculty development and feedback to improve the learning environment and faculty performance [7]. Some medical schools have designated educational data and analytics support personnel who collect and analyze student feedback about faculty performance, while others rely upon individual clerkships or departments to develop their own methods for monitoring faculty performance [8]. Although many medical schools collect student surveys about faculty teaching, published details about content, response rates, ease of use/acceptance, use in clinic-based education, and the impact upon faculty and students is sparse [9, 10] and often mixed with overall curriculum, not individual faculty performance. This report describes our psychiatry clerkship’s experience of developing a well-received pilot for an individualized faculty performance assessment system that sets the foundation for a more individualized multi-specialty school-wide faculty performance and improvement program.

The Uniformed Services University of the Health Sciences (USUHS) psychiatry clerkship and the School of Medicine (SoM) did not have a system for collecting student feedback for individual faculty. Previously, the psychiatry clerkship utilized a simple open-ended response satisfaction survey with prompts such as, “What are we doing right?” and “What would you suggest we improve?” and asked students to name faculty who are exceptional and faculty who need constructive feedback via open-comment items that were often left unfilled. Simultaneously, the SoM utilizes an all-specialty end-of-rotation survey focused on the rotation learning climate and clerkship curriculum that includes open-ended prompts “Please name any faculty member(s) from this clerkship whom you would like to recognize for excellence/needs improvement in teaching and briefly describe why,” also often left unanswered. Neither the previous psychiatry clerkship nor the SoM end-of-rotation surveys contained prompts about specific faculty or their behaviors.

There was a clear need to develop standardized, focused student feedback/assessment tools that allow students to evaluate each of their assigned faculty’s performance based upon the student’s observations and experiences with them. To address this, the psychiatry clerkship developed a behaviorally anchored “Student Assessment of Faculty Performance” (SAFP) tool modeled on the existing “Faculty Assessment of Student Performance” (FASP) tool used for individual student assessment by the clerkship faculty. This educational case report aims to describe the development, content, administration, and acceptance of this new tool as part of the psychiatry clerkship’s new clinical faculty performance review program.

## Reforming Clerkship Faculty Evaluations

The authors developed a behaviorally anchored SAFP tool in conjunction with faculty, student, and SoM Dean input. The SAFP mirrors the behaviorally anchored psychiatry clerkship FASP tool that faculty use to assess individual student performance at the end of each rotation [11]. Annually, the SAFP data from all students are aggregated for each faculty into the Individual Faculty Aggregated Student Assessment of Faculty Performance (IFA-SAFP) report. Additionally, the individual faculty evaluations of students (their evaluation/grading practices) data are collected from the FASP and aggregated into the Individual Faculty Grading Profile (IFGP). Both are consolidated into the Combined Faculty Grading Profile and Performance Report (CFGPP) and distributed to each faculty on an annual basis. The psychiatry clerkship utilizes end-user satisfaction surveys distributed to students and faculty to obtain input about the ease of use and the impact of the new program. Specifics about the content, deployment timelines, and tracking of the collected feedback are discussed below.

### Evaluation Tools

#### Student Assessment of Faculty Performance Tool

The SAFP tool (available for review/use from the corresponding author) is completed by students to assess faculty performance. Each survey includes eight behaviorally anchored multiple-choice questions about an individual faculty’s performance in the areas of student engagement, punctuality, preparedness, articulating expectations and providing feedback, working relationships with students, working relationships with colleagues/staff/teams, patient interactions, and fund of knowledge. Each of these includes demonstration of leadership and modeling as part of the highest rated behavioral anchor. One Likert-scale item asks if the faculty fostered an inclusive learning environment with respect for learners from all backgrounds, and two questions ask students to rank the faculty member’s teaching/mentorship skills and fund-of-knowledge from their own perspective in comparison to all other clinical faculty they have ever worked with on a scale from lowest 25% to top 5%. Examples of these prompts are provided in Table 1. Selections are weighted 1 through 4 (or 6) upon collection (respondents do not see the weight of each selection) in ascending order. Any selection at the lowest level for the behaviorally anchored questions is recorded as a “red flag” and is reviewed upon receipt by the clerkship coordinator and referred to the clerkship director for attention.

**Table 1:** SAFP behaviorally anchored questions

Prompt	Choices
Regarding student engagement, my faculty^b^:	…did not spend any time with me during the rotation^a^ …spent time with me only in relationship to direct patient care (i.e. tasking to do interviews, notes) …spent time with me in relationship to direct patient care and regularly offered clinical teaching points based on patient cases …spent time with me in relationship to direct patient care, offered clinical teaching points, and also provided appropriate mentorship and modeling to become a successful medical professional
Regarding preparedness, my faculty^b^:	…was habitually/always unprepared for the day/meeting^a^ … was occasionally prepared for the day … was usually prepared for the day/meeting, … was usually well-prepared for the day/meeting and discussed/expected the same from all team members
Regarding articulating expectations and providing feedback to me, my faculty^b^:	… was routinely unresponsive/too busy or hostile to requests to provide feedback and/or never articulated rotation expectations^a^ … sometimes struggled to provide time for feedback, rarely expressed expectations … developed and offered feedback that was helpful when requested and routinely provided clear expectations … actively and regularly discussed expectations and created opportunities for feedback, incorporating it effectively in a manner that impacted my performance positively
Compared to all other clinician-faculty you have worked with, regardless of clerkship specialty, how do you rate this faculty’s teaching skills and medical mentorship of clerkship students?^c^	1. Bottom 25% 2. Below average 3. Top 50% 4. Top 25% 5. Top 10% 6. Top 5%

^a^This selection has a reflex required open box comment and required email to clerkship director

^b^This same format is repeated for questions regarding faculty punctuality

^c^This same format is used for a question regarding clinical skills/demonstrating and providing patient care

The SAFP is anonymously completed by clerkship students for each of their three assigned faculty via a link sent by the clerkship student management system on the last day of each 5-week rotation. The system has a reciprocal feature allowing students to “pre-view” their faculty’s assessment of the student’s performance once they complete the SAFP. If they choose to not complete the SAFP, they forfeit early access to that faculty’s assessment of their own performance and must wait until they receive the complete clerkship evaluation document at the end of the grading period. Faculty members have no access to the individual student SAFPs. The clerkship director has access to the individual SAFP data no sooner than the close of the grading period 6 weeks after each rotation.

#### Faculty Assessment of Student Performance Tool

Faculty assess student clerkship performance with the behaviorally anchored FASP, adapted from the reporter, interpreter, manager, educator (RIME) model [12]. The FASP has behaviorally anchored prompts in the areas of professionalism, reporting, interpreting, and management. The student’s final clerkship assessment includes an average of 3 FASPs for the student’s final behavioral anchor scores, and all open comments made by each faculty are included in their final assessment.

#### Individual Faculty Grading Profile

The IFGP is an individualized, aggregated report of faculty site demographics, specific faculty student supervision load, and a summary of the FASP selections for all students they evaluated that year. This report includes averaged evaluation selections in each area of the clerkship made by all faculty generated in the same academic year. Group data and individual data are provided to assist faculty in recognizing the assessment practices of their peers, and to allow annual review of outlying assessment practices with themselves, their site directors, and clerkship leadership.

#### Individual Faculty Aggregated Student Assessment of Faculty Performance

The IFA-SAFP includes an individualized, aggregated, and anonymized report of their clerkship SAFP data and all open feedback comments for each faculty member for the preceding academic year.

#### Combined Faculty Grading Profile and Performance Report

The IFA-SAFP are combined as the CFGPP, individually delivered to all clerkship faculty after the academic year. Faculty are provided with the report via email and informed that it is for their own use and not shared with their organization’s rating or performance review system. They are encouraged to use the information for their own professional development, to discuss their results with their site director and the clerkship director, and to consider including the reports for academic promotion purposes.

#### End User Surveys

To determine SAFP ease of use and perceived utility of this program, anonymous, voluntary end-user satisfaction surveys are delivered to all students immediately after rotation completion, and to faculty after the first individualized reports are delivered at the end of the academic year.

### Early Expansion Beyond Psychiatry

The family medicine clerkship joined this project after the first year as an initial expansion to the SoM. Their clerkship has many more clerkship sites and rotating faculty due to their outpatient focus, and they determined that they could not use the psychiatry clerkship/SoM student management system. Their SAFP is completed by clerkship students at the end of their rotation only about the performance of their assigned site directors. The family medicine tool has identical content but is delivered via a survey tool that does not have the reciprocal feature for the student to “pre-view” their own evaluation by the faculty once they complete their SAFP. The family medicine clerkship requires the completion of the SAFP, while the psychiatry clerkship does not. The family medicine clerkship has not deployed end-user surveys.

## Responses and Outcomes

Psychiatry SAFP and FASP data have been collected for all rotations of 2022 and 2023. Family medicine SAFP data has been collected for academic year (AY) 2023 (see Table 2). The SAFP response rate was a remarkable 97.5% for Psychiatry and 91% for Family Medicine. All (100%) of psychiatry clinical faculty received their CFGPP each year, with 62 receiving two annual reports to date.

**Table 2:** Collected assessments and responses

Collection data	AY 2022 Psychiatry	AY 2023 Psychiatry	Total Psychiatry	AY 2023 Family Medicine*n*
Students (#)	169	183	352	180
SAFP assigned to students (#)	505	543	1048	Not assigned
SAFP self-selected by students (#)	N/A	N/A	Not self-select	360
Returned SAFP (#/%)	493 (98)	528 (97)	1021 (98)	326 (91)
Faculty evaluated by students (#/%)	87 (100)	92 (100)	117 (100)^a^	120^b^
FASP completed (#)	505	543	1048	N/A
CFGPP/IFA-SAFP to faculty	87	92	179^a^	120^c^
Faculty end survey received (#/%)	41 (47)	8 (9)	49	N/A
Student end survey received (#/%)	71 (42)	95 (5)	166 (48)	N/A

*SAFP* Student Assessment of Faculty Performance, *FASP* Faculty Assessment of Student Performance, *IFA-SAFP* Individual Faculty Aggregated Student Assessment of Faculty Performance, *CFGPP* Combined Faculty Grading Profile and Performance Report

^a^62 faculty taught in both years, receiving 2 reports

^b^Family Medicine students assess site directors only

^c^Psychiatry sends the CFGPP; Family Medicine sends only the IFA-SAFP

The end-user faculty satisfaction survey response rate for AY 2022 was 47% (*n* = 41), where 83% of the respondents agreed that the individualized reports were useful. Some faculty asked for yearly comparisons to make it easier to see if there is improvement. For AY 2023, only eight end-user surveys were received due to a technical error and were not analyzed due to the small error-biased sample size.

Interestingly, 16 faculty emailed the clerkship coordinator upon receipt of their annual report, all with comments of appreciation and thanks, as well as the following: “Now I am even more motivated, and energetic, to teach these stellar students,” “I really appreciate these as a way to reflect on my areas for further growth/development,” “I am honored to be involved in teaching the best medical students in our country,” and “I like the feedback, good job.”

End-user student survey response rates for AY 2022 and 2023 were 42% and 52%, respectively. Eighty-six percent (86%) of student respondents agreed that the SAFP could be completed in a reasonable amount of time. Three-fourths (75%) agreed that the SAFP was easy to understand and felt comfortable giving honest feedback to their assigned faculty. Two-thirds (63%) felt they gave constructive and actionable feedback through the tool. Just over half (53%) believed their feedback would help improve faculty members’ performances as educators and thought that the SAFP would benefit clerkships other than psychiatry.

## Discussion and Future Directions

Building a new faculty performance program for our psychiatry clerkship has had its technical difficulties, but student acceptance and completion have been robust for both psychiatry and family medicine (98% and 91%, respectively); the difference for psychiatry is likely due to having assigned faculty and a reciprocal “pre-view” option in the student management system. These return rates far exceed our SoM and clerkship return rates of the past despite different distribution and data collection approaches.

Our initial experiences during the first 2 years of program development have been shared at our university curriculum meetings and at national educational meetings, emphasizing the acceptance and response rate of the new tools [8, 13–15]. Our institution has expanded the project to include the family medicine clerkship using a different platform for distribution and collection of their SAFP tool and is now working to develop a faculty feedback and performance “dashboard” to improve access and provide more timely information. The psychiatry faculty data are now continuously being collected in hopes that analysis across multiple years, individual faculty, and clerkship sites will determine if there is any change or impact upon student assessment of faculty performance and/or faculty assessment of students over time.

In conclusion, the authors made successful inroads in developing user-friendly tools to collect and distribute individualized faculty clinical teaching performance and their individual student grading data to 100% of the psychiatry clerkship faculty on an annual basis, expanded the use of some of these tools in other specialties, and have determined that the SAFP is well received and has a high return rate. Future improvements would include a thematic analysis of the student open-comment responses in the SAFP over a longer period, as well as assessing whether there are changes to the way that faculty evaluate the students and are evaluated by them over a longer period. Semi-structured focus-group interviews may be more effective in soliciting feedback about the assessment of faculty performance tools and processes instead of end-user surveys of all clerkship students and faculty. Sharing future findings about the program with students and faculty may also improve student belief that providing this feedback is of benefit. The authors share the experience of developing and delivering a new faculty performance program in hopes of providing useful insights and lessons learned about providing student input for every clerkship faculty member utilizing behaviorally anchored tools.

## Data Availability

N/A.

## Declarations

The opinions and assertions expressed herein are those of the author(s) and do not reflect the official policy or position of the Uniformed Services University of the Health Sciences or the Department of Defense.
